# Multi-Omics Techniques for Soybean Molecular Breeding

**DOI:** 10.3390/ijms23094994

**Published:** 2022-04-30

**Authors:** Pan Cao, Ying Zhao, Fengjiao Wu, Dawei Xin, Chunyan Liu, Xiaoxia Wu, Jian Lv, Qingshan Chen, Zhaoming Qi

**Affiliations:** 1 College of Agriculture, Northeast Agricultural University, Harbin 150030, China; caopp2022@outlook.com (P.C.); tianshi198937@126.com (Y.Z.); wfjfwl@126.com (F.W.); xdawei@163.com (D.X.); cyliucn@126.com (C.L.); 2 Department of Innovation, Syngenta Biotechnology China, Beijing 102206, China

**Keywords:** soybean, multi-omics, molecular breeding

## Abstract

Soybean is a major crop that provides essential protein and oil for food and feed. Since its origin in China over 5000 years ago, soybean has spread throughout the world, becoming the second most important vegetable oil crop and the primary source of plant protein for global consumption. From early domestication and artificial selection through hybridization and ultimately molecular breeding, the history of soybean breeding parallels major advances in plant science throughout the centuries. Now, rapid progress in plant omics is ushering in a new era of precision design breeding, exemplified by the engineering of elite soybean varieties with specific oil compositions to meet various end-use targets. The assembly of soybean reference genomes, made possible by the development of genome sequencing technology and bioinformatics over the past 20 years, was a great step forward in soybean research. It facilitated advances in soybean transcriptomics, proteomics, metabolomics, and phenomics, all of which paved the way for an integrated approach to molecular breeding in soybean. In this review, we summarize the latest progress in omics research, highlight novel findings made possible by omics techniques, note current drawbacks and areas for further research, and suggest that an efficient multi-omics approach may accelerate soybean breeding in the future. This review will be of interest not only to soybean breeders but also to researchers interested in the use of cutting-edge omics technologies for crop research and improvement.

## 1. Introduction

Soybean [*Glycine max* (L.) *Merr*.] originated in China over 5000 years ago; China currently ranks third in soybean production worldwide, and total production has increased to meet market demands [1]. In 1830, soybean travelled the great distance to Europe by the “ancient silk road”, and from there it spread throughout the world to North America, South America, India, and elsewhere [2].

At that time, soybean breeders were mainly farmers, brewers, or suppliers, and they selected soybeans based on their experience and market demands [3]. Records show that artificial hybridization was performed in the early 1900s [4], and breeders continued to advance soybean breeding through cooperation with cell biologists and molecular geneticists in the late twentieth century [5].

Soybean is a major oil crop that also provides plant protein to the food industry. Through genetic modification of its fatty acid composition, soybean oil has been tailored to meet end-user needs more successfully than other conventional oils. Three modified oils are already commercially available. Oils with a linolenic acid (18:3) content from 1% to 8% can reduce or eliminate the need for chemical hydrogenation to achieve the stability and shelf life required for certain food applications. The elimination of fatty acids produced by chemical hydrogenation and trans-hydrogenation is important for human health. An increase in oleic acid (18:1) from 25% to >80% also increases oil stability and shelf life. Reduction in palmitic acid (16:0) from 11% to <4% produces low levels of saturated fatty acids and is very beneficial for cardiovascular health [6]. Soybean meal extracted from high-oleic-acid soybeans is rich in protein and inhibits the growth of colon, liver, and lung cancer cells [7].

**Figure 1:**
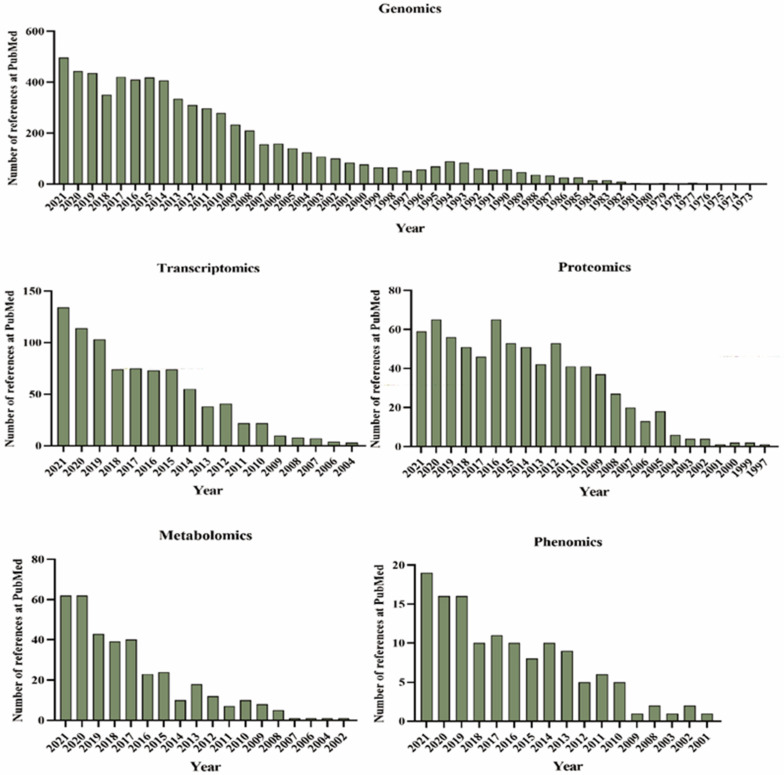
Numbers of PubMed references for different types of omics techniques.

In response to global demands for different soybean product profiles, quantitative trait locus (QTL) mapping laid the foundation for breeding and selection of major traits. The SoyBase database was first established in the 1990s as the USDA Soybean Genetics Database, which collected genetic resources for soybean, including genetic maps and information on Mendelian genetics (www.soybase.org (accessed on 22 January 2022)). With efforts from many groups and researchers, the first soybean reference genome (Williams 82) was released in 2010, marking a new era in soybean omics research [8]. Studies of genomics, transcriptomics, proteomics, metabolomics, and phenomics have increased dramatically in recent years (Figure 1). The regulatory network of soybean is extremely complex; although single-omics approaches can reveal or explain specific biological phenomena, their results may be difficult to apply directly to soybean breeding. Additionally, now, given the huge amounts of omics data available, significant thought must be given to the best means of using them efficiently. In this review, we comprehensively describe the latest progress in crop omics techniques and discuss ways that soybean breeding may be further improved using multi-omics information in the future.

## 2. Multi-Omics Research Progress

### 2.1. Soybean Genomics Research Progress

After the release of the soybean reference genome, Lam et al. (2010) sequenced 31 wild and cultivated soybean genomes and identified genetic diversity patterns and signatures of selection [9]. The genome sequence of *Glycine soja* (*G. soja* var. IT182932) was also subsequently reported.

Because of at least two whole genome duplication (WGD) events in the soybean genome over the last 60 million years, it is quite common for genes to be present in two or four copies [10]. A more accurate reference genome can accelerate functional genomics research, and in 2018, Shen et al. [11] reported the de novo assembly of the Chinese soybean “Zhonghuang 13” genome based on advanced sequencing technologies, including single-molecule real-time (SMRT) sequencing, Hi-C chromosome conformation capture, and optical mapping. This genome exhibited more than 250,000 structural variations compared with Williams 82. Subsequently, the genome of the wild soybean accession W05 was assembled, with a size of 1013.2 Mb and a contig N50 of 3.3 Mb [12]. The Williams 82 genome was reassembled and reannotated (version Wm82.a4.v1), integrating ~1.6 million ESTs and 1.5 billion paired-end Illumina RNA-seq reads with homology-based gene predictions [13].

A pan-genome from seven wild soybean relatives was reported in 2014, facilitating the construction of a core genome and enabling genomic comparisons to identify lineage-specific genes and genes with copy number variations or large-effect mutations [14]. Recently, a graph-based pan-genome was constructed from 26 representative wild and cultivated soybeans based on long-read sequencing, enabling the detection of numerous genetic variations that could not be identified from short-sequence reads alone [15]. The pan-genome not only provides complete information on the entire soybean genome, but also serves as a platform for investigating the evolution and functional genomics of soybean [16,17,18].

Recent studies have discovered a number of genes with wide applicability to soybean production. In 2016, Zhao et al. [19] performed genetic and molecular studies of flowering genes by crossing early maturing soybean varieties. The hybrid offspring segregated for two maturity loci, *E1* and *E9*, and detailed molecular analysis of the *E9* locus was performed to identify the causal gene. Fine mapping, sequencing, and expression analysis indicated that *E9* was *FT2a*, a homolog of *Arabidopsis FLOWERING LOCUS T*. The recessive allele of *E9/FT2a* delays flowering through reduced transcript abundance due to allele-specific transcriptional inhibition, associated with the sore-1 insertion. Therefore, *FT2a* transcript abundance is directly related to changes in soybean flowering time. The *E9* allele can maintain vegetative growth in an early-flowering genetic background and can also be used as a long-juvenile allele, delaying flowering under short-day conditions at lower latitudes. Soybean is the main leguminous crop in temperate regions, and the photoperiod response is a key factor in its latitude adaptation. The varieties introduced into low-latitude, temperate regions mature earlier and have very low yields. The introduction of long-juvenile (*LJ*) traits extends the vegetative stage and increases yield under short-day conditions, thereby enabling the expansion of tropical planting. In 2017, Lu et al. [20] used natural variation in the soybean *J* locus to improve adaptability to tropical areas and increase yield.

The composition and content of fatty acids in soybean seeds are very important for the quality of soybean oil. Fatty acids are the main products of oil biosynthesis and substrates of oil catabolism, and they are an important energy source for organisms. In a 2020 study aimed at increasing soybean oil content, Wang et al. [21]. reported strong selection on *GmSWEET10a* during soybean domestication based on resequencing data from more than 800 genotypes. Selection on *GmSWEET10a* not only increased soybean seed size and oil content but also reduced protein content. These results were validated using near-isogenic lines with haplotype substitution and transgenic studies. Wang et al. also found that *GmSWEET10b* and its homolog *GmSWEET10a* are functionally redundant and are under selection in breeding, making the *GmSWEET10b* allele a good target for soybean breeding. Research on *GmSWEET10a* and *GmSWEET10b* has shown that the transport of sucrose and hexose contributes to sugar distribution from seed coat to embryo, thereby determining oil and protein content and seed size in soybeans. Selection on the *GmSWEET10a* allele is thought to have promoted the initial domestication of a variety of soybean seed traits, and targeted selection of the superior allele *GmSWEET10b* may further improve the yield and seed quality of modern soybean varieties.

In summary, although only a few genes suitable for use in soybean breeding have been identified through genomics analysis to date, the *J* locus and flowering-time-related genes show promise for moving soybean planting areas into new latitude locations [19,20]. Seed oil and protein content genes could also be used to improve soybean seed composition [21]. As the number of sequenced wild and cultivated soybean germplasm resources increases, there is an increasing need for a worldwide data integration platform that combines a series of reference genomes, a pan-genome database, and basic analysis platforms, expanding the scope of use of a wide range of available soybean germplasm resources.

### 2.2. Soybean Transcriptomics Research Progress

With the development of sequencing technology, transcriptomics is rapidly becoming a mainstay of plant science research [22,23,24]. Transcriptome analysis with next-generation sequencing, also known as RNA sequencing (RNA-seq), enables unbiased, high-throughput detection of all expressed transcripts. This technology offers new insights into molecular profiles and signaling pathways at the level of systems biology and can identify useful gene markers for the efficient breeding of soybean. Recent RNA-seq data provide an overall picture of the metabolic activities of storage compounds during soybean seed development and enable modeling of the gene network associated with seed lipid and protein deposition. During soybean seed filling, the embryo develops as carbohydrates, oils, and proteins are stored in the cotyledons [25,26,27,28]. Severin et al. [29] (2010) performed transcriptome sequencing of different tissues and different developmental stages of Williams 82 soybean, then constructed an RNA-seq atlas of hierarchically clustered gene expression profiles; highly expressed genes and legume-specific genes were also identified. Subsequently, similar research documented transcript patterns during soybean seed development and identified seed-specific genes and expression patterns [30,31].

Genes encoding storage proteins (e.g., beta-conglycinin and glycinin) and enzymes of lipid or starch synthesis were most highly expressed at the embryonic stage when fresh weight was highest, suggesting that these storage compounds are immediately deposited into seeds before initiation of the desiccation process. Other genes expressed at the dry seed stages have been annotated as water-deficit-associated hydrophilic proteins [32], including dehydrins and late-embryogenesis-abundant (LEA) proteins that facilitate the preservation of nutrients and cellular structures during seed desiccation. More significantly, several transcription factors (TFs) have been identified as major regulators of seed development, including the *APETALA2 (AP2), VIVIPAROUS1/ABI3-LIKE (VAL), FERTILIZATION INDEPENDENT ENDOSPERM* (FIE) [33], *GLABRA2 (GL2), PICKLE (PKL)* [34], and DNA-binding-with-one-finger (DOF4) transcription factors [35]. PKL is an important activator of embryonic development, and FIE inhibits premature endosperm development. VAL1 and VAL2 [36] regulate the transition from embryonic development to germination. Seed filling development is also regulated by *ABSCISIC ACID (ABA)-INSENSITIVE 3 (ABI3), LEAFY COTYLEDON 2 (LEC2), FUSCA3 (FUS3), and WRINKLED1 (WRI1) TFs* [26,37]. These TFs may influence the deposition of carbohydrates, oils, and proteins during seed filling. The interactions among TFs during embryonic development and germination are relatively complex and involve several TFs at each stage. Identification and characterization of transcript polymorphisms in soybean lines of different oil compositions provided evidence that mutation of FAD2-1A and FAB2C influenced oleic acid and stearic acid levels, respectively, in elite soybean lines [38]. Jang et al. [39] (2015) analyzed the gene expression patterns of seed protein and oil synthesis during early soybean seed development. Luet al. [40] (2016) built gene co-expression networks based on 40 transcriptome datasets from developing seeds of cultivated and wild soybean accessions, and they identified the two hub genes GA20OX and NFYA. Qi et al. [41] (2018) performed RNA-seq of four chromosomal segment substitution lines (*CSSLs*) that differed in seed storage composition, identifying seven hub genes through the integration of meta-analysis and RNA-seq co-expression networks. Yang et al. [42] (2018) screened out the three hub genes *GmABI3b*, *GmNFYA*, and *GmFAD2-1B* through dynamic transcriptome analysis of developing soybean seeds. Recently, as RNA sequencing technology has advanced, additional hub genes such as LEC2, ABI3, and SWEET10a have been identified by RNA-seq and co-expression analysis [43]. These datasets and hub genes provide additional resources and candidate gene lists for functional validation.

Transcriptome analysis has also provided an in-depth understanding of the molecular and genetic responses that underlie soybean adaptation to environmental stresses [44,45,46,47]. Transcriptomic data can be extremely valuable for examining differences in gene expression between stress-tolerant and stress-sensitive genotypes, facilitating the development of stress-tolerant genotypes, which is one of the primary objectives of soybean breeding. Comparative transcriptome analysis of soybean exposed to different stresses led to the identification of functional and regulatory genes that act in response to individual and combined stresses, enabling their use in breeding for combined stress tolerance. These genes comprise protein kinases, phosphatases, and a number of TFs from the basic helix–loop–helix (bHLH), ethylene response factor (ERF), myeloblastosis (MYB), no apical meristem (NAC), and WRKY families [48]. Changes in the expression of genes encoding osmolyte-regulating enzymes, aquaporins, LEA proteins, chaperone proteins, and reactive oxygen species (ROS) scavengers, which maintain ionic balance by active transport and protect cell membrane integrity, were also found to be associated with various stress responses in soybean. Transcriptomic studies permit the comparative genomic analysis of cultivated crops and their wild relatives, enabling the identification of additional target genes that are crucial for the improvement of breeding processes. For example, RNA-seq has been used to examine the expression profiles of wild soybean species under alkaline stress [49], providing insight into the functions of alkaline-stress-responsive genes and the molecular basis of wild soybean alkalinity tolerance [50].

Transcriptomics research is a high-throughput approach that uses large-scale datasets to study the overall transcript levels of multiple genes that function in a biological process. With rapid development of high-throughput sequencing technology, further reductions in sequencing costs, and continuous improvements in large-scale data processing capabilities, transcriptomics has become a common experimental approach for solving biological problems through the discovery of new transcripts, development of detailed transcriptional atlases, and accurate identification of metabolic pathways [51]. In addition, with the continuous development of sequencing technology, the current analysis methods and basic assumptions need to be reevaluated and adjusted in order to better cope with the large amount of omics data in the future [24]. At the same time, cell- and tissue-specific transcriptomics technology has been used in specific biological applications, such as plant transformation [52]. Single-cell transcriptomics is continuing to mature, providing precise spatial and temporal insights into biological processes [53,54]. To date, transcriptomics research has identified only a few genes that can be used in soybean breeding, but continued integration of transcriptomics with genomics and other omics approaches could help breeders to optimize soybean regulatory networks and refine hub gene candidates for further soybean breeding.

### 2.3. Soybean Proteomics Research Progress

Proteins are the source of biological phenomena, the executors of physiological functions, and the direct embodiment of biological activities. Proteomics, the study of protein expression and function, has developed rapidly since its inception, and ever-increasing amounts of proteomic information, together with rapidly expanding plant genome resources and EST sequence libraries, have provided help for the identification of proteins. Proteomics provides the necessary basis for the study of soybean protein expression [55]. In recent years, it has been widely used for research on various aspects of soybean biology, such as growth and development, stress, and root–nodule interactions, and the resulting studies have deepened our understanding of changes in protein expression during the soybean life cycle [56,57,58,59,60,61].

Before 2012, soybean proteome studies relied heavily on 2D gel electrophoresis [62], but advances in liquid chromatography with tandem mass spectrometry (LC–MS/MS) have made high-throughput proteomics possible, promoting greater efficiency and accuracy in proteome research. Hajduchet al. [63] (2005) constructed high-resolution proteome reference maps of seed filling in soybean. Subsequently, the integration of two-dimensional gel electrophoresis, semicontinuous multidimensional protein identification technology (Sec-MudPIT), and LC–MS has improved our understanding of the metabolic processes that occur during seed filling in soybean [64].

Afroz et al. [56] (2011) analyzed the proteome profiles of leaves, hypocotyls, and roots of young soybean seedlings and detected tissue-specific proteins. Since then, proteomics has increasingly been used to analyze enzyme expression and regulatory mechanisms involved in accumulation during seed storage [57]. Root hairs and developing seeds have been analyzed using isobaric tags for relative and absolute quantitation (iTRAQ), and specific proteins related to root hair and seed development have been identified [58,59].

Seed oils and seed storage proteins are the main seed storage reserves in soybean. Xu et al. [60] (2015) used proteomics to analyze differences in global protein expression profiles and oil synthesis between a high-oil soybean cultivar (Jiyu 73, JY73) and its parents. Proteomics has also been widely used in stress biology to identify key proteins. For example, Xu et al. [61] analyzed *GmDGAT1-2* transgenic soybeans with high oil content using quantitative proteomics and lipidomics. They showed that *GmDGAT1-2* overexpression induces downregulation of lipoxygenase and upregulation of oleosin, thereby significantly altering total fatty acid composition. A proteomics approach also revealed flood and drought response mechanisms in soybean. Wang et al. [65] (2018) identified sensitive tissues of stressed soybean at different developmental stages based on protein profiles, documenting the stress responses of young plants and seedlings exposed to combined stresses in a tissue-specific manner. In 2017, Wang et al. [66] performed tissue-specific proteomics studies of soybean seedlings under flooded conditions, and Wang et al. [67] (2021) demonstrated the dual effect of calcium on soybean radicle protrusion using quantitative proteomics.

Recently, Islam et al. [68] (2019) performed quantitative proteomic analysis of low-linolenic-acid transgenic and control soybean seeds. They revealed perturbations in proteins related to fatty acid metabolic pathways, including a lower abundance of proteins associated with FA initiation, elongation, and desaturation processes and with β-oxidation of α-linolenic acids. Wei et al. [69] (2020) combined quantitative proteomics with physiological data and revealed the effects of temperature and humidity stress on cotyledon, embryo, leaf, and pod vigor in soybean.

Although the breadth of proteome research in soybean is still lower than that in other crops, it nonetheless provides a starting point for functional genomics studies of natural product biosynthesis mechanisms and biotic and abiotic stresses in soybean [55,56,57,58,59,60,61]. The information obtained from proteomics can help to identify novel proteins, determine the expression patterns of their corresponding genes, and enable their molecular cloning. Soybean research could be further advanced by the construction of a soybean proteome reference map. A combination of proteomics, genomics, and transcriptomics could enable the screening of elite alleles and the development of molecular markers, providing new possibilities for soybean molecular breeding. Recent advances in protoplast and sequencing technologies have enabled single-cell transcriptomic studies in plants, but single-cell proteomics will need to develop much further to achieve a comparable throughput. A major problem with single-cell proteomics is its inherently low sample volume that challenges traditional sample preparation protocols and the sensitivity of current liquid chromatography–mass spectrometry (LC-MS) systems [70]. Nonetheless, protein data arguably reflect the execution and control of most cellular processes more closely than transcriptome data, and efficient, high-throughput techniques for analyzing protein expression, interactions, and modifications will be essential for understanding the molecular mechanisms that underlie plant phenotypes [71].

### 2.4. Soybean Metabolomics Research Progress

Metabolomics is a new approach to the qualitative and quantitative analysis of small metabolites with relative molecular weights <1000 in a given tissue or cell. It is an important aspect of systems biology, and its development will have implications for future soybean research. Metabolomics analyses can reveal specific metabolic signaling pathways, providing key resources for gene discovery, metabolic engineering, and the elucidation of regulatory mechanisms. Quantitative metabolomics techniques for the detection of plant metabolites include liquid chromatography–electrochemistry–mass spectrometry (LC–EC–MS), gas/liquid chromatography–mass spectrometry (GC/LC–MS), thin-layer chromatography (TLC), Fourier transform infrared (FT–IR) spectroscopy, NMR, direct infusion mass spectrometry (DIMS), and capillary electrophoresis–LC–MS [72,73,74,75,76]. The LC–MS, GC–MS, NMR, and capillary electrophoresis MS techniques are most commonly used in plant metabolomics [77,78,79]. Compared with genomics, transcriptomics, and proteomics, the results of plant metabolomics techniques are more directly related to the plant phenotype. The identified metabolites have the dual effect of influencing or regulating both gene transcription and protein expression. Subtle changes that occur at other levels of regulation can be further amplified at the metabolome level [80]. The general process of metabolomics studies involves plant sample collection, metabolite isolation, and the detection of metabolite type, content, and status using assay techniques to construct a metabolic fingerprint [81]. This is combined with bioinformatics analysis to mine relevant information and integrate metabolic pathways for a comprehensive understanding of plant metabolic processes and metabolite changes [82].

Single-cell mass spectrometry provides information on metabolite abundance in cell populations, enabling the identification of hidden phenotypes, metabolic states, and rare cells. Comparing gene expression, protein function, and metabolite levels in individual cells could provide a comprehensive understanding of cellular physiology. In recent years, the combination of MS with novel sampling and ionization techniques has emerged as an important tool for single-cell metabolomics [83]. MS-based single-cell metabolomics enables the simultaneous detection of multiple metabolites from a single cell without preselection or labeling, thus mapping phenotypes at the single-cell level. Although this approach is still relatively new, it has been adopted by a growing number of active research groups who are developing cell sampling and ionization techniques, data analysis tools, and applications to answer important biomedical and environmental questions [84].

Characterizing mechanisms of metabolic regulation is crucial for modifying soybean seed composition. Using a nontargeted metabolomics approach, 169 metabolites were identified from mature seeds of 29 representative soybean cultivars and then mapped onto a metabolic network. These metabolites were mainly involved in key pathways of seed development, such as the tricarboxylic acid cycle, glycolysis, amino acid biosynthesis and catabolism, nitrogen utilization, antioxidant utilization, lipid oxidation, and secondary metabolite accumulation [85]. Significant variations in metabolite abundance and clear metabolite–metabolite correlations among different soybean cultivars were also demonstrated. The isoflavone profiles of soybean germplasms highlighted the diverse varieties of isoflavones present in soybean [86], and several aglycones were associated with different levels of shade tolerance at the seedling stage [87]. The effects of seed dry weight, seed coat color, and maturity on metabolite abundance have also been determined [88,89]. For instance, black soybean seeds were used to investigate the effect of maturity on metabolite abundance at different maturity stages. Several metabolites showed different responses to seed maturation, and the isoflavone content was markedly related to seed maturity. In addition, plant metabolites that change through specific pathways can enhance the nutritional value of genetically modified soybean by promoting the accumulation of isoflavones in developing seeds.

The use of metabolomics techniques was evaluated in a comprehensive study to explore the results of abiotic stress on soybean metabolites. The results showed that exposure of soybean plants to abiotic stress increased the biosynthesis of secondary metabolites, among which glycine and proline act as major osmoprotectants, playing important roles in the reduction in osmotic damage induced by abiotic stress. Levels of different polyphenols (hydroxycinnamates and flavonoids), phenylpropanoids, alkaloid caffeate, and phytochemicals (daidzin, daidzein, syringic acid, formononetin, genistin, and genistein) also increased [90], all of which are known to respond to plant drought stress. A metabolomics approach was recently used to discover metabolic markers applicable to crop improvement. This concept was first introduced by an earlier study that identified metabolites with high predictive values as biomarkers for plant biomass accumulation and plant breeding improvement. The role of these markers in crop enhancement was verified by recent studies [91], and their potential for use in soybean selection was also evaluated. Recent studies compared changes in metabolites between tobacco and soybean after exposure to drought, and levels of 4-hydroxy-2-oxoglutaric acid in tobacco roots and pinitol and coumestrol in soybean roots were markedly increased, suggesting that these may be useful markers for differentiating lines grown under well-watered conditions from those exposed to drought stress [92].

In the last decade, metabolomics studies have opened up new horizons for the elucidation of plant metabolic pathways and genetic architecture. Metabolomics studies have resolved the isoflavone profile of soybean [84], identified metabolites associated with dry weight and maturity of soybean grains [87], and greatly accelerated the differentiation of disease-resistant from disease-susceptible soybean varieties under stress conditions [93]. However, because plants have complex metabolic pathways and diverse mechanisms of product synthesis, metabolomics is still in its infancy, and gaps remain between metabolomics findings and practical breeding applications. There is no single metabolomic analysis method that can cover all metabolites [94]. Methods of metabolite isolation, identification, and data analysis used for different research objectives are also different and have strict requirements for sample processing techniques. The research performed in soybean to date has focused mainly on known metabolites, and the large number of unknown metabolites obtained without appropriate structural information and identification impedes further studies and applications. Metabolomics also lacks deep integration with other approaches, making it a relatively narrow area of research. It remains difficult to effectively obtain comprehensive information on plant metabolites, and resolving these methodological issues will be a breakthrough for future metabolomics research.

### 2.5. Soybean Phenomics Research Progress

Given the rapid development of sequencing technology and the sheer number of plant materials to be tested, the collection of phenotypic information by appropriate high-throughput phenotyping technologies is particularly important for plant breeding. However, plant phenotypes are complex and dynamic, and they are easily affected by the environment. Manual investigation of plant phenotypes is characterized by low efficiency and large errors [95]. As an approach to solve these practical problems, plant phenomics has begun to receive significant attention [96]. At present, there are only 132 published articles related to high-throughput phenotyping in soybean, and 120 have been published in the past decade. The number of studies is increasing year by year, showing that breeders are becoming increasingly aware of the importance of accelerating soybean breeding through phenotyping studies.

Plant growth and development follow a strict growth regime, but harvested plants differ in their cotyledon size, resistance to stress, and metabolic capacity. Small developmental differences can result in dramatic changes in both physical traits and internal characteristics of plants [97]. Plant phenomics leverages high-throughput, high-resolution phenotyping technologies and platforms to acquire phenotype data during and after plant production [98]. It is characterized by the large amounts of trait data acquired and the ability to divide the same trait into multiple smaller traits for testing. Data acquisition with fixed, quantitative, and uniform acquisition standards facilitates high-throughput automated analysis, enhancing the accuracy of crop phenotype identification and further promoting the efficiency of plant breeding and cultivation management [99].

For example, professional unmanned aerial vehicles (UAVs) can be equipped with high-definition dual-camera multi-spectral equipment and can obtain accurate soybean yield estimates and efficient pod maturity classifications by reconstructing time course multispectral high-throughput image data [100]. Fusion of high-spatial-resolution RGB, multispectral, and thermal data from UAV systems has improved the estimation accuracy of soybean physiological, biochemical (e.g., chlorophyll content, nitrogen concentration), and biophysical parameters (e.g., leaf area index, aboveground fresh and dry biomass) [101]. The color and texture features of early-season RGB images of the soybean canopy have also been used to predict soybean yield, maturity, and seed size [102]. Professional UAVs equipped with expensive multispectral, hyperspectral, and thermal imaging equipment are increasingly used for high-precision sampling of soybean canopy traits, such as height, area, temperature, and leaf wilting [103,104,105]. However, it is difficult to obtain higher-dimensional phenotypic traits from 2D images, and some estimates of some morphological traits still require calibration. To this end, researchers have performed three-dimensional reconstruction of plant morphology from two-dimensional image sequences using fully open-source structure from motion (SFM) and multi-view stereo (MVS) approaches [96]. For example, plant height and growth phenotype data can be obtained by establishing high-density 3D point clouds from plant image data [106]. In 2020, researchers used 3D reconstruction technology to analyze the “phenotypic fingerprint” and growth pattern of soybean plants throughout the growth period [107]. This cost-effective 3D reconstruction method can replace expensive laser scanners, with the potential to automate some procedures.

The use of high-throughput phenotyping to record plant responses to stress can enable the identification of resistant plants and the discovery of new genes [108]. When studying the biological effects of stress, a combination of high-throughput phenotyping ground vehicles, unmanned aerial systems, and digital images taken with smartphones can be used to automatically assess the severity of iron deficiency chlorosis (IDC) in real time [109], enabling field screening for large-scale soybean IDC tolerance [110]. Zhou et al. developed an automated plant phenotyping system in an established greenhouse and used it to analyze chlorophyll content and salt tolerance by continuously photographing and extracting image features with digital cameras (red, green, and blue), thereby demonstrating its feasibility [111]. Soybean is sensitive to flooding stress. Researchers used five-band multispectral and infrared thermal imaging cameras to extract canopy image features from three flight altitudes and then used deep learning to estimate soybean flooding damage scores [105]. By measuring such phenotypes, *QTLs* or genes related to abiotic stress can be located, and new soybean varieties with strong stress resistance can be cultivated.

With the development of remote sensing, robotics, visualization, and artificial intelligence, plant phenomics research has entered a stage of rapid growth. However, the huge amounts of data and numbers of pictures also present unprecedented challenges. Machine learning, the foundation of artificial intelligence whereby algorithms make predictions and learn from data without explicit programming, has been used in attempts to solve this problem [112]. Deep learning, a new field within machine learning, was originally proposed in 2006 [113]; it now includes approaches such as convolutional neural networks (CNNs), multilayer perceptrons (MLPs), and recurrent neural networks (RNNs) [114]. CNNs have been most widely applied to plant phenotyping, and commonly used CNN deep learning frameworks include TensorFlow [115], PyTorch [116], and Caffe [117]. At the same time, the development of deep learning has been supported by advances in cloud computing and GPU parallel computing. At present, there is a high demand for modern, high-frequency, multi-site, standardized phenotype acquisition, and the standardization and storage of phenotype data is a current subject of concern. Nonetheless, deep learning and low-cost sensors have been applied to a number of image-based tasks with impressive results [114].

For example, machine learning, multi-modal data fusion, and deep learning have been applied to soybean multi-sensor data from UAV systems to enable accurate prediction of soybean yield [118,119]. Riera et al. developed a multi-view-image-based fusion architecture for monitoring soybean pods and estimating yield through deep learning and demonstrated its effectiveness [120]. In another study, a novel high-throughput image analysis method was developed to rapidly determine and analyze the morphology and color of 39,065 soybean seeds from 400 lines [121]. Algorithms such as random forests and deep CNNs are well suited to many vision-based computer problems. For example, a system based on an image processing algorithm could detect materials other than grain (MOGs) in harvested soybeans on a large scale [122]. UVA-based multispectral images combined with a random forest approach enabled estimation of soybean maturity in different lines [106]. Compared with traditional image analysis methods, CNN is simultaneously trained end-to-end without an image feature description and extraction process, and it has been used to efficiently segment single soybean seeds and calculate their morphological parameters [123,124]. Soybean phenotype data include not only aboveground information but also belowground root phenotype data, and the fully automated soybean nodule acquisition pipeline (SNAP) combines RetinaNet and UNet deep learning architectures for nodule detection and segmentation. Compared with traditional methods, SNAP reduces the labor and inconsistency associated with nodule calculations and enables earlier assessment of the effects of genetic and environmental factors and their interactions on nodules [125].

These methods enable timely, efficient, and accurate prediction of soybean phenotypes in different regions and scales, revealing the regional differentiation and evolution of soybean phenotypic traits and assisting with soybean breeding and cultivation decisions.

The main soybean phenotypic traits currently investigated by phenomics include yield [126], maturity [102], leaf area index [101], plant height [106], cotyledon size, color [121], and response to abiotic stresses [105,109,111]. Image analysis techniques include fluorescence imaging [127], thermal imaging [128], two- or three-dimensional color imaging [129], and infrared spectral imaging [130]. Despite recent advances in plant phenomics research, the study of plant phenotypes has been limited mainly to the description of external physical traits and has largely failed to address internal and biochemical characteristics, hindering its application to practical breeding. In addition, there are many issues that require further research. Phenotyping equipment is costly and expensive, and although it greatly reduces labor, it still requires personnel with a specific biological and technical background who can follow a standardized process. Some of the equipment can be operated only under strict environmental and weather conditions, and large-scale field data collection can be subject to large deviations due to weather conditions, particularly for spectral equipment, which requires high light levels. Some cutting-edge technologies for crop phenotyping, such as artificial intelligence techniques and CT imaging, have rarely been used in soybean breeding. In the context of the big data era, the information obtained by machine learning is a huge multidimensional matrix. It will be important to help breeders filter useful information from such massive datasets and integrate this information with other biological data in order to ultimately perform deep data mining for the selection of new plant varieties.

## 3. Molecular Breeding in Soybean

Food security is among the most important topics for human society. Plant breeding, as a major approach to increasing the food supply, is one of the oldest agricultural practices in human civilization. To date, there have been three major innovations in plant breeding: artificial selection, cross breeding, and molecular breeding. A fourth innovation, optimization and precision design breeding, is also underway [95]. Molecular breeding refers to the application of molecular biology techniques to breeding, i.e., breeding at the molecular level, and it is the most widely used breeding approach at present.

Long ago, our ancestors began to domesticate wild plants. They selected desired phenotypes and individuals from among the plants they cultivated and began to intentionally control plant reproduction, ushering in the first stage of plant breeding, artificial selection. Breeding for selected phenotypes continued for thousands of years through the 19th century, when Mendel’s law was first proposed in 1865 [131]. After that, pedigree breeding was developed on the basis of segregation, and plant breeding entered the second stage, hybridization. The structure of DNA was discovered in 1953, and life science entered the molecular era [132]; on this basis, molecular biology techniques were developed to perform breeding at the molecular level. These techniques currently include marker-assisted selection [133] and transgenic breeding [134]. Molecular marker-assisted breeding (marker-associated breeding) is based on the close linkage between molecular markers and genes that determine target traits. By detecting specific molecular markers, the presence of target genes can be determined, enabling the selection of target traits. This approach is rapid, accurate, and free of interference from environmental conditions. Transgenic breeding uses genetic engineering to produce new varieties with desired characteristics through the introduction of specific genes. These methods have transformed plant breeding from pure phenotypic selection to a combination of genotypic and phenotypic selection, enabling the production of new varieties that meet human requirements.

Since the assembly of the first plant genome in *Arabidopsis thaliana* 20 years ago, developments in high-throughput sequencing technology have provided reference genomes for more than 800 land plants. High quality reference genomes and large amounts of genotyping data provide great convenience for quantitative genetic analysis of complex traits.

Experiments with the obtained genes have increased the oil content of soybeans, increased the content of soybean oleic acid, and reduced the content of linolenic acid. In 2010, Pham [135] et al. found that three polymorphisms in the FAD2-1B allele of two soybean lines resulted in missense mutations. The mutant FAD2-1B allele was associated with an increase in oleic acid content. Pham [136] et al. also found that high-oleic-acid soybeans could be obtained by combining the mutant FAD2-1A and FAD2-1B genes. However, despite their high oleic acid content, these soybeans still contained 4–6% linolenic acid, which may be enough to cause oxidation instability in the oil. Therefore, one or two mutated FAD3 genes were added into a high-oleic-acid background to further reduce linolenic acid content [137]. Recently, a high-oleic-, low-linolenic-acid soybean variety with elevated vitamin E content was developed by molecular-assisted breeding [136]. Oleic acid levels affect human health, and producing high quality soybeans with high contents of oleic acid and other health-promoting components should continue to be the focus of further research. Molecular-assisted breeding has also been used to improve carbohydrate profiles [136], pod shatter [138,139], yield and latitude adaptation [19,20,140], and seed oil and protein content [21] in soybean breeding.

Soybean molecular breeding involves the integration of multiple disciplines. With progress in science and technology, simple integration of theories from individual disciplines is no longer sufficient for the continued development of soybean molecular breeding. Although only a few genes identified from omics approaches have been used in soybean breeding to date, the integration of one to four genes into a new variety has been achieved with the assistance of genomics tools. Nonetheless, continued improvements in soybean breeding will be dependent on advances in multiple omics. In addition to the traditional omics methods mentioned above, new omics techniques have also emerged, including metagenomics, single-cell omics, and various types of epigenetic omics. These new omics methods provide more data for the characterization of complex traits. Nonetheless, it remains a challenge to integrate different omics approaches into an overall omics strategy to dissect the detailed connections among different omics datasets and further our understanding of complex trait regulation.

Crop production is increasingly difficult owing to water shortages, climate change, and extreme weather events caused by the current global environment. Therefore, new innovations in precision design breeding (i.e., Stage 4.0 breeding) will be required to feed the growing world population. The development of new technologies, especially the development of cross-disciplines based on economics, has brought Stage 4.0 breeding to the forefront. A prerequisite for precision design breeding is the accurate association of genotype with phenotype. Therefore, it is necessary to determine the genetic anatomy of agronomic traits and identify the corresponding genotypic variations. In the past 40 years, multiple revolutions in DNA sequencing technology have significantly improved sequencing throughput and quality, and sequencing costs have continued to decline, greatly promoting functional genomics research [95,141]. Nonetheless, the challenge of integrating large, dissimilar datasets remains.

## 4. Further Perspectives

With the growth of the human population, continuous improvements in living standards, and the potential threat posed by global environmental change to food production, it is necessary to perform intensive research into the molecular mechanisms of high and stable crop yields under adverse conditions. The excellent alleles and germplasm resources of China have been established independently. A design innovation system for environmental adaptive breeding has been established in China and combined with big-data climate modeling and accurate predictions to produce high-quality, stable crop yields and provide important guarantees for national food security and people’s life and health [95,142].

**Figure 2:**
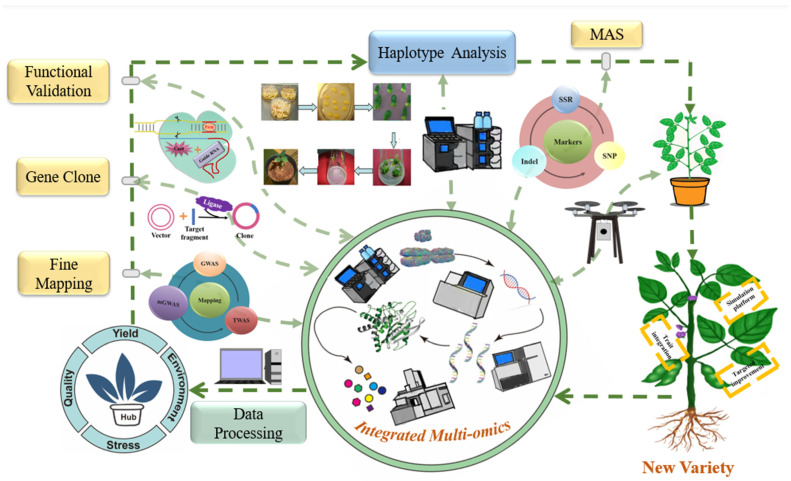
Multi-omics approaches for soybean molecular breeding.

Here, we propose a conceptual workflow for the use of multiple omics datasets to identify key factors that regulate soybean yield, seed quality, stress biology, and other factors. First, wild and cultivated soybean reference genomes, pan-genome databases, and analysis platforms can be expanded to include a wider range of soybean germplasm resources in the future. The integrative analysis of multi-omics data can be used to construct the regulatory networks of individual traits, analyze the synergistic relationships among traits, and improve our overall understanding of molecular regulatory networks. The comprehensive use of genetics, multi-omics, molecular biology, and other technical approaches can identify key regulatory genes involved in relevant pathways and clarify their effects. To obtain further details, genome-wide association studies (GWAS), transcriptome-wide association studies (TWAS), metabolite-GWAS (mGWAS), and population genetics can enable fine mapping of a hub gene in natural germplasm populations or segregation populations. The hub gene can then be cloned to create overexpression or knock-out plants for phenotype validation. Multiple omics strategies can accelerate the identification of the hub gene and can provide a better understanding of its regulatory network. During the hub gene screening process, identification of excellent alleles and further utilization of genomic resources from wild soybean are also important future directions. Germplasm with excellent alleles can be selected for crossing with the main cultivated variety. With the assistance of marker development for the hub gene(s) and a high-throughput phenotyping system, important hub genes can be integrated into the molecular design of a new soybean variety by high-throughput molecular-assisted breeding selection (Figure 2). The sharing of information and resources from different groups should be strengthened worldwide, and hub genes and their excellent alleles can be used to accelerate the development of soybean breeding [142,143].

## 5. Conclusions

Multiple omics approaches show promise for the efficient improvement of soybean breeding in future research. The genome is the basic foundation of soybean germplasm, whereas the transcriptome, proteome, metabolome, and phenome are the upper layers. In soybean breeding practices, genomics, transcriptomics, proteomics, metabolomics, and high-throughput phenotyping will need to be better integrated to construct the regulatory networks of complex traits and efficiently identify hub genes. Genome editing of specific hub genes will also help with their functional validation. After marker development for the hub gene(s), important or specific hub genes can be integrated to design a new soybean variety. Further development of integrated multi-omics resources can promote the efficient and accurate discovery of excellent alleles, providing more possibilities for soybean breeding.

## 6. Supplementary Research Methodology

Keywords for each omics technique were used for literature searches at PubMed (https://pubmed.ncbi.nlm.nih.gov/ (accessed on 22 January 2022)); search terms included soybean genome, soybean transcriptome, soybean proteome, soybean metabolome, soybean high-throughput phenotype, and soybean multi-omics. All literature on each technique was collected as completely as possible, and we focused on milestone literature on soybean and literature published in the most recent 3–5 years. We developed the present review from these collected references, summarizing the research questions, important data, research methods, and major results of each paper.

## Data Availability

Not applicable.
